# Architectural and Mechanical Changes after Five Weeks of Intermittent Static Stretch Training on the Medial Gastrocnemius Muscle of Active Adults

**DOI:** 10.3390/sports11040073

**Published:** 2023-03-27

**Authors:** Morgan Lévenéz, Matthieu Moeremans, Cédric Booghs, Florent Vigouroux, Clément Leveque, Walter Hemelryck, Costantino Balestra

**Affiliations:** 1 Environmental, Occupational, Aging (Integrative) Physiology Laboratory, Haute Ecole Bruxelles-Brabant (HE2B), 1160 Brussels, Belgium; 2 Anatomical Research and Clinical Studies, Vrije Universiteit Brussel, 1090 Brussels, Belgium; 3 DAN Europe Research Division (Roseto-Brussels), 1160 Brussels, Belgium; 4 Physical Activity Teaching Unit, Motor Sciences Department, Université Libre de Bruxelles (ULB), 1050 Brussels, Belgium

**Keywords:** muscle fascicle, passive torque, pennation angle, stiffness, hysteresis, ultrasonography

## Abstract

We investigated the effects of intermittent long-term stretch training (5 weeks) on the architectural and mechanical properties of the muscle–tendon unit (MTU) in healthy humans. MTU’s viscoelastic and architectural properties in the human medial gastrocnemius (MG) muscle and the contribution of muscle and tendon structures to the MTU lengthening were analyzed. Ten healthy volunteers participated in the study (four females and six males). The passive stretch of the plantar flexor muscles was achieved from 0° (neutral ankle position) to 25° of dorsiflexion. Measurements were obtained during a single passive stretch before and after the completion of the stretching protocol. During the stretch, the architectural parameters of the MG muscle were measured via ultrasonography, and the passive torque was recorded by means of a strain-gauge transducer. Repeated-measure ANOVA was applied for all parameters. When expressed as a percentage for all dorsiflexion angles, the relative torque values decreased (*p* < 0.001). In the same way, architectural parameters (pennation angle and fascicle length) were compared for covariance and showed a significant difference between the slopes (ANCOVA *p* < 0.0001 and *p* < 0.001, respectively) suggesting a modification in the mechanical behavior after stretch training. Furthermore, the values for passive stiffness decreased (*p* < 0.05). The maximum ankle range of motion (ROM) (*p* < 0.01) and the maximum passive torque (*p* < 0.05) increased. Lastly, the contribution of the free tendon increased more than fascicle elongation to the total lengthening of the MTU (ANCOVA *p* < 0.001). Our results suggest that five weeks of intermittent static stretch training significantly change the behavior of the MTU. Specifically, it can increase flexibility and increase tendon contribution during MTU lengthening.

## 1. Introduction

Static stretch training is an effective method to increase the range of motion (ROM) and decrease muscle stiffness [1,2]. In athletic settings, static stretching is usually applied to prevent sport injuries. However, the effects of stretching on the structural properties of muscles and tendons remain unclear, probably due to differences in methodological approaches, such as training duration, the number and duration of weekly stretching sessions, intermittent versus continuous static stretching, and stretch intensity [3]. For an updated very recent review with a multi-level meta-analysis about the chronic effects of static stretching exercises on muscle strength, power, and flexibility, we recommend the excellent manuscript of Arntz et al. [4].

Usually, ROM is used to quantify changes in flexibility. However, it can be influenced by various factors such as pain, stretch tolerance, and reflex activation of the agonist muscle [5,6]. Another useful method is to determine the joint torque during submaximal passive stretch [7,8], which is often used in conjunction with variations in joint angle to characterize the viscoelastic properties of the human muscle–tendon unit (MTU) in vivo. Recently, it has been confirmed that a high-intensity static stretching program is more effective for increasing ROM and decreasing muscle stiffness than a low-intensity program [2]. An intermittent stretching approach also seems to be more effective for increasing ROM and changing the mechanical properties of the musculotendinous complex [9].

Although researchers agree that the limitations of this lengthening are both neurological and mechanical [6,10,11], the mechanical factors remain ambiguous and disputed. Weppler and Magnusson [12] pointed out that numerous studies tried to explain the lengthening of the stretched muscle as a consequence of changes in mechanical properties (such as viscoelastic deformation, plastic deformation, or neuromuscular relaxation).

Many previous studies showed that ultrasonography is a valid analysis method and can be used for the viscoelastic properties of the stretched muscle, namely the stiffness and hysteresis of the human muscle–tendon structures in vivo [13,14,15,16]. Moreover, this non-invasive method allows for the characterization of changes in fascicle length and tendon tissue behavior during stretching [17,18,19,20,21]. This approach is of interest since the relative contribution of muscle and tendon structures to the total MTU lengthening remains disputed. Herbert and al. [17] reported a tendon contribution to the MTU lengthening of up to ≈75%, while a study by Morse and al. [20] observed a relatively equal contribution of muscle length and the free tendon.

Some previous studies reported no significant changes in the muscle architecture of medial gastrocnemius (MG) after 4 to 12 weeks of static stretch training [1,2]. However, others reported a modification in the muscle architecture of MG after 6 weeks of a machine-assisted static stretching program [22] or 8 weeks of high-intensity static stretching in the biceps femoris [23]. In addition, few authors investigated the effects of stretching on MTU considering both muscle and tendon. Nevertheless, Kubo and al. [24] showed that stretch training can specifically affect the viscosity of tendon structures.

To analyze these changes, the aim of this study was two-fold: on the one hand, to investigate the in vivo effects of five weeks of intermittent static stretch training protocol on the human MTU viscoelastic and architectural properties of the MG; and, on the other hand, to assess the relative contribution of muscle and tendon structures to the total MTU lengthening. We hypothesize that five weeks of intermittent static stretch training could alter MTU viscoelastic and architectural properties since this approach seems to be more effective for increasing ROM and changing the mechanical properties of the musculotendinous complex. Therefore, we realized this original protocol, using a slow and progressive passive lengthening of the MTU to avoid inducing the myotatic reflex [25,26], at various ankle joint angles and following the suggestion of Nakamura et al. [27].

## 2. Materials and Methods

### 2.1. Participants

Ten healthy subjects (four females and six males) volunteered for this study, after approval from the Bio-Ethical Committee for Research and Higher Education, Brussels (No. B200-2023-030), and written informed consent was obtained. All experimental procedures were conducted in accordance with the Declaration of Helsinki [28]. The participants aged 22.9 ± 3.2 years (height: 175.7 ± 6.9 cm and body mass: 71.3 ± 9.5 kg; means ± SD). Height and weight were measured using a fixed stadiometer and an electronic scale (Tanita DC360S), respectively. All the subjects were healthy physiotherapy students and were engaging in physical activity on average for two hours a week. The physical activity organized during the period of the experiment was the sport training proposed by their physical education professor and consisted of running in the forest once a week during the 2 h of their required physical education curriculum. All the subjects were instructed on the experimental procedure and avoided strenuous physical activity the day before the experiment. Exclusion criteria consisted of signs or a history of neuromuscular disorders or traumatic lesions.

### 2.2. Experimental Setup and Protocol

**Figure 1:**
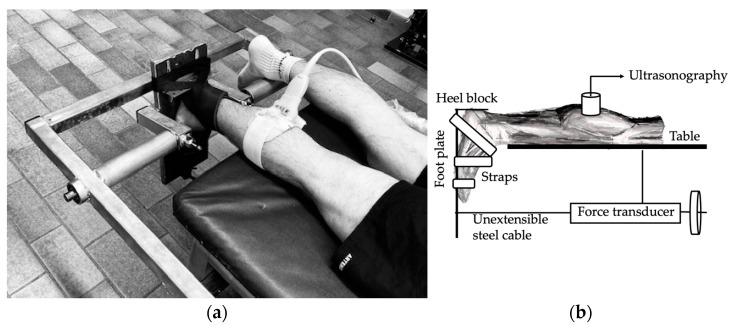
(**a**) Experimental setup with the volunteer lying prone on a table, with both legs extended and one foot secured to a footplate by straps and an adjustable heel block; (**b**) schematic illustration showing the location ultrasound probe and the force transducer.

A prone position was used for each subject along with straps at the ankles and an adjustable heel block. Both legs were extended, and one foot was secured to a footplate by straps (Figure 1). Extra foam was applied underneath the knee as needed to ensure full extension. The angular displacement of the ankle joint was monitored by means of a domestically made and calibrated linear potentiometer that was mounted on the rotational axis of the footplate. The sole of the foot was perpendicular to the tibial axis in control conditions (0°; perpendicular to the anatomical axis). An unextensionable cable connected the footplate to a sliding mechanical device that measured passive ankle dorsiflexion by passively dorsiflexing the ankles from 0° to 25° (increment of 5° steps), at an angular velocity of 2.5°/s. Subjects were instructed to relax during the stretching phase of the experiment, and not to resist the footplate’s movement. To reach total ROM, the angle was not limited to 25°, but for viscoelastic measurements, this angulation was the maximum taken. All of our participants were instructed to reach their own perceived level of maximum stretch within the pain limit during testing and exercise. The supervision of the exercises within the laboratory (except on weekends) made it possible to optimize each stretching session.

The torque produced by the plantar flexor muscles during the passive stretch was recorded by means of a strain-gauge transducer (U2000 Load Cell, Sherborne Sensors Limited, Basingstoke, UK). The passive torque values for all dorsiflexion angles were expressed in absolute and relative values. The slope of the passive torque–angle curve from 15° to 25° was used to estimate passive stiffness for each subject. The hysteresis loop produced by the loading and unloading curves of the passive torque, as a function of the angle of dorsiflexion, allows the determination of the amount of dissipated elastic energy. The area under the loading and unloading curves represents, respectively, the elastic energy input and the elastic energy available.

The ultrasound machine was a Sonosite M-Turbo (FUJIFILM Sonosite Inc., Amsterdam, The Netherlands), the apparatus settings were adapted for musculoskeletal measurements, and a linear probe model HFL 50× was used that has a width of X cm and a frequency range from 15 to 6 MHz. This ultrasound machine was used to investigate the architectural changes in the MG muscle during the stretching. The probe was fixed into the leg (at 30% of the distance between the center of the medial femoral condyle and the center of the medial malleolus), over the mid-belly of the muscle. After identifying the muscle fascicle, a custom-made molding resin (Aquaplast™) sheath was strapped to the skin to hold the echographic/ultrasound linear probe in position. By maintaining constant orientation and pressure, the sheath ensured the probe’s integrity. Transmission gel was used to make acoustic contact with the probe. Commercial software (PCTV vision) was used to acquire images on a personal computer and analyze them offline.

### 2.3. Training Protocol

Each subject followed a passive stretch training protocol for 5 weeks. Subjects were asked to stretch their gastrocnemius muscle for 30 s, 3 times a day, for 5 weeks. The stretches were performed at the maximum degrees of dorsiflexion possible while avoiding crossing the individual pain threshold. The stretched leg was maintained in a straight position, and the hip was positioned in neutral rotation. The subjects were asked to perform the classic wall stretch (legs in a split stance). While facing the wall with one foot forward and the other foot behind, hands were placed flat on the wall at chest height. Keeping the rear leg straight and the foot flat on the floor with the front leg knee bend, they would lean forward, hold for 30 s, and slowly release and switch sides. Warm-up was intentionally not performed before the stretch training. Stretching was supervised by laboratory members and was carried out in the laboratory before class, during the lunch break, and in the evening. No monitoring was carried out on weekends.

### 2.4. Data Analysis

**Figure 2:**
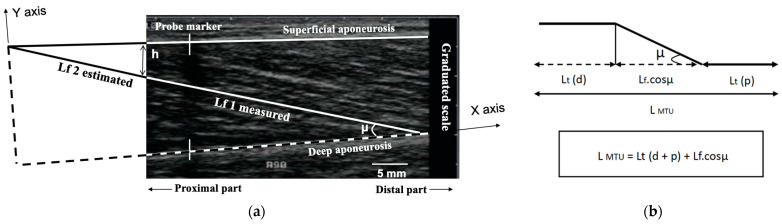
(**a**) Ultrasound image obtained along the medial longitudinal axis of the medial gastrocnemius (MG) muscle. Muscle fascicles can be identified as the oblique striations occurring between superficial and deep aponeurosis. When the end of the fascicle extended off the acquired ultrasound image, the total fascicle length was estimated via trigonometry (total Lf = lf 1 measured + lf 2 estimated = lf 1 + (h/sinμ)) by assuming a linear continuation of the fascicles. Pennation angle (µ) corresponds to the angle between the deep aponeurosis and the fascicle; (**b**) schematic illustration of the length of the muscle–tendon unit (L_MTU_) comprising the sum of the distal (L_t_ (d)) and proximal (L_t_ (p)) lengths of the tendon and the component of the muscle along the horizontal axis (L_f_.cosµ).

Two parameters were measured from each MG ultrasound scan: muscle fascicular length (Lf), and pennation angle (µ) [21,29,30,31]. The muscle fascicle was defined as a clearly visible fiber bundle lying between the superficial and deep aponeuroses, identified and marked by using an image program (ImageTool). The pennation angle (μ) was determined as the angle between the fascicle and its insertion on the deep aponeurosis. The fascicle length (Lf) was measured along the marked fibers’ bundle, from the superficial to the deep aponeurosis. When the end of the fascicle extended off the acquired ultrasound image, fascicle length (Lf) was estimated using trigonometry (total Lf = lf 1 measured + lf 2 estimated = lf 1 + (h/sinμ)) [10,32,33] by assuming a linear continuation of the fascicles (Figure 2a). The total muscle–tendon unit length (L_MTU_) at rest (ankle angle at 0°) was determined through the use of a tape measure over the skin, after having identified the proximal (medial femoral condyle) and distal (superior edge of the calcaneum) insertions of the MG. All measures were performed by the same experienced operator (M.L.) with more than 100 scans/year, which is recommended to maintain competency. In our laboratory, the mean intraobserver variability for muscular measurement for the operator (M.L.) recorded on the same day, the same site, and the same subject was 0.8 ± 0.2%.

Concerning the estimation of the change in L_MTU_ during the stretch, the position of the ultrasound probe over the MG impeded a direct measure of the L_MTU_ change during the stretching exercise. As a result, we used the regression equation provided by Grieve et al. [34] to calculate the change in L_MTU_ during stretching: ∆L = −22.185 + 0.30141 (90 + A) − 0.00061 (90 + A)^2^, where ∆L is the change in L_MTU_ due to the change in dorsiflexion angle (A) (Figure 2b). To estimate the lengthening of the tendon (Lt; distal and proximal parts) during the stretch, the change in Lf along the longitudinal axis (Lf.cosµ) of the MTU was subtracted from the respective change in L_MTU_, at each angle of ankle dorsiflexion (Figure 2b) [10,17].

### 2.5. Statistical Analysis

Conventional statistical methods were used for calculating the means, standard deviations (SD), and standard error (SE). The Kolmogorov–Smirnov and the D’Agostino-Pearson Omnibus tests were used to assess normality. Changes in passive torque, pennation angle, and fascicle length were analyzed by means of a repeated-measure analysis of variance (ANOVA). A Bonferroni post hoc test was used to identify the significant differences among the means. The specific time points before and after stretch training were analyzed by means of a Student’s *t*-test, or Mann–Whitney test when appropriate. The best-fitting relations were tested with linear regressions using the least-square method. The linear regressions (pennation angle and fascicle length) recorded before and after the stretch training were compared using an analysis of covariance (ANCOVA). Power calculation was performed a priori for repeated-measure ANOVA (effect size = 0.67, alpha error = 0.05, power = 0.80) using G*Power calculator 3.1 software (Heinrich Heine University, Düsseldorf, Germany); the requisite number of participants for this study was more than 8. The ANOVA effect size was evaluated with Cohen’s term d and classified as follows: small (d = 0.2), medium (d = 0.5), and large (d ≥ 0.8). For all analyses, the level of significance was set at *p* < 0.05. Data are reported as means ± SD in the text.

## 3. Results

### 3.1. Changes in Passive Torque after Completion of the Stretch Training Protocol

Before stretch training, as the angle of dorsiflexion increased from 0° to 25°, the passive torque increased exponentially (y = 5.7 * e(0.068 * x); r^2^ > 0.98) from 3.5 ± 1.2 Nm at 0° (neutral position) to 30.4 ± 10.4 Nm at 25° of dorsiflexion.

**Figure 3:**
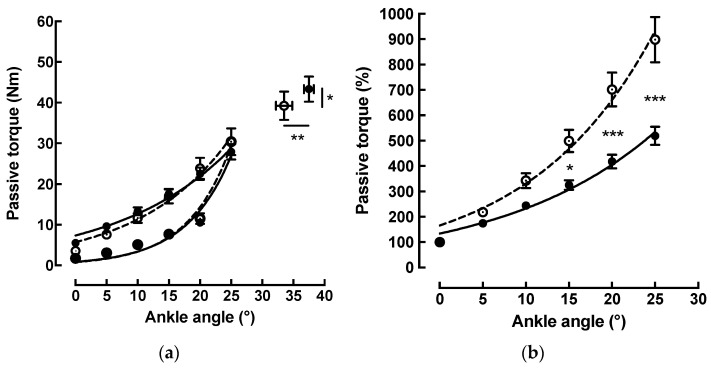
(**a**) Change in the passive torque (Nm) produced by the plantar flexor muscles as a function of the ankle angle before (---○---) and after (―●―) stretch training. Curves represent the loading and unloading curves forming a hysteresis loop. Isolated data correspond to the maximum ankle range of motion and the corresponding mean maximum passive tension before (○) and after (●) stretch training. Data are means ± SE for 10 subjects. * *p* < 0.05; ** *p* < 0.01. (**b**) the correlation between relative passive torque (%) and ankle angle was best fitted by the following exponential equations before (---○---) and after (―●―) stretch training: y = 165.5 * e ^0.069x^ (r^2^ = 0.98) and y = 133.6 * e ^0.055x^ (r^2^ = 0.98). Data are means ± SE for 10 subjects. * *p* < 0.05; *** *p* < 0.001.

After the training, the same trend was observed. Once again, the passive torque increased exponentially (y = 7.3 * e(0.054 * x); r^2^ > 0.98) from 5.5 ± 1.2 Nm at 0° (neutral position) to 27.9 ± 6.2 Nm at 25° of dorsiflexion (Figure 3a).

To compare the differences in the passive torque values at any dorsiflexion angle between the pre-training and post-training tests, the relative passive torque was calculated as a percentage for all dorsiflexion angles (Figure 3b). The analysis of each individual curve showed a nonlinear increase before (y = 165.5 * e(0.069 * x); r^2^ > 0.98) and after (y = 133.6 * e(0.055 * x); r^2^ > 0.98) stretch training. The ANOVA analysis revealed a stretching effect for the passive torque calculated as a percentage (d ≥ 0.8). The comparison of those curves showed a decrease in the relative passive torque values after the stretch training at 15° (*p* < 0.05), 20° (*p* < 0.001), and 25° (*p* < 0.001). There was a significant increase in the maximum ankle ROM from 33.5 ± 4.1° to 37.5 ± 2.6° after training (+13%; *p* < 0.01) and the maximum passive torque from 39.3 ± 11.2 Nm to 43.4 ± 9.8 Nm (+13.2%; *p* < 0.05) (Figure 3a).

Ankle passive stiffness reached an average value of 1.34 ± 0.5 Nm/° before stretch training and an average value of 1.04 ± 0.4 Nm/° after. The comparison between pre- and post-stretch training showed a significant decrease of −21.6% (78.4 ± 19.1%; *p* < 0.01). After the training, the dissipated elastic energy, represented by the hysteresis loop, decreased by −61.2% (38.8 ± 18.6%; *p* < 0.001).

### 3.2. Changes in MG Architecture after Completion of the Stretch Training Protocol

**Figure 4:**
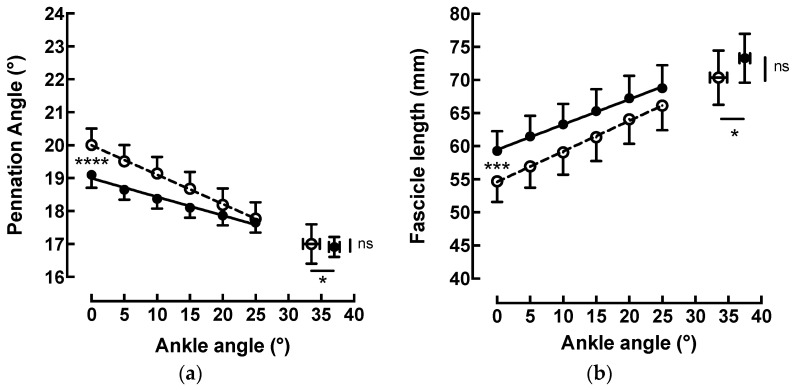
(**a**) Change in the linear relation between the pennation angle (°) and the ankle angle (°) before (y = −0.089x + 20; r^2^ > 0.99; ---○---) and after (y = −0.056x + 19.00; r^2^ = 0.98; ―●―) stretch training. (ANCOVA *p* < 0.0001). Data are means ± SE for 10 subjects. * *p* < 0.05; **** *p* < 0.0001; (**b**) change in the relation between the fascicle length and the ankle angle (°) before (y = 0.46x + 52.61; r^2^ > 0.99; ---○---) and after (y = 0.38x + 59.47; r^2^ > 0.99; ―●―) stretch training. (ANCOVA *p* < 0.001). Data are means ± SE for 10 subjects. * *p* < 0.05; *** *p* < 0.001.

Before the stretch training, the pennation angle (Figure 4a) decreased linearly (y = −0.089x + 20; r^2^ = 0.99) from 20.0 ± 1.56° at 0° (neutral ankle position) to 17.8 ± 1.5° at 25° of dorsiflexion (−11.2% ± 2.1%). Lf (Figure 4b) increased linearly (y = 0.46x + 54.61; r^2^ = 0.99;) from 54.7 ± 9.9 mm at 0° (neutral ankle position) to 66.1 ± 11.7 mm at 25° of dorsiflexion (+21.1 ± 4.1%).

Similar observations were made after the training. The pennation angle decreased linearly (y = −0.056x + 19.00; r^2^ = 0.98) from 19.1 ± 1.1° at 0° (neutral ankle position) to 17.6 ± 0.8° at 25° of dorsiflexion (−7.5% ± 1.4%). Lf increased linearly (y = 0.38x + 59.47; r^2^ = 0.99; *p* < 0.0001) from 59.3 ± 9.5 mm at 0° to 68.8 ± 11.0 mm at 25° of dorsiflexion (+16.0 ± 3.9%). Statistical analysis of the pre- and post-test results of the linear regression of the pennation angle and fascicle length showed an extremely significant difference between the slopes (ANCOVA *p* < 0.0001; d = 0.80 and *p* < 0.001; d = 0.47, respectively).

### 3.3. Estimation of the Relative Changes in Length after Completion of the Stretch Training Protocol

Length of myotendinous unit (L_MTU_): The average LMTU increased from 396.5 ± 26.7 mm at 0° (neutral ankle position) to 414.0 ± 27.9 mm at 25° of dorsiflexion (+17.5 ± 1.2 mm; +4.4%).

**Figure 5:**
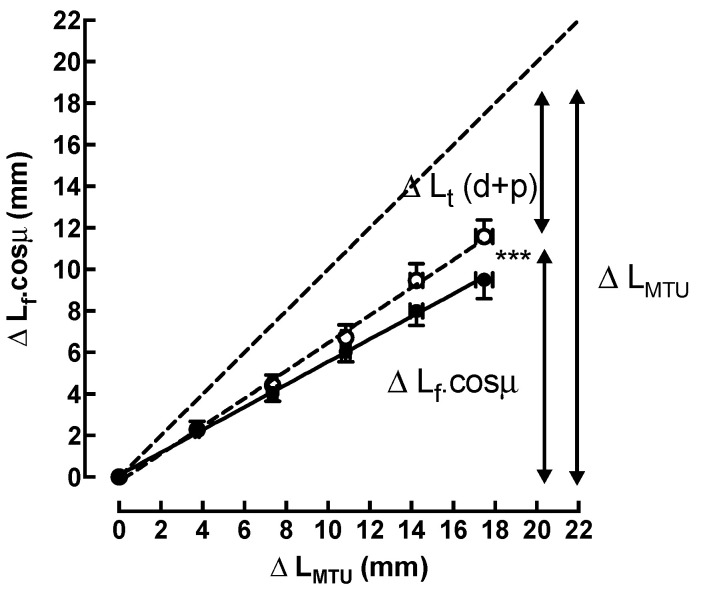
Relationship between the longitudinal lengthening fascicles (ΔL_f_.cosµ) and the lengthening of the muscle–tendon unit (ΔL_MTU_) during passive stretching from 0° to 25° (dorsiflexion) of ankle angle before (---○---) and after (―●―) stretch training (ANCOVA *p* < 0.001 ***). The length of the muscle–tendon unit (L_MTU_) corresponds to the sum of the distal (Lt (d)) and proximal (Lt (p)) lengths of the tendon (Lt (d + p) and the component of the muscle along the horizontal axis (Lf.cosµ). Uppercase delta (Δ) means “change” or “variation”. Data are means ± SE for 10 subjects.

Lf.cosµ and Lt: Statistical analysis of the pre- and post-test results showed a different behavior (ANCOVA *p* < 0.001; *d* = 0.77) in the relative contributions of the free tendon, and of the fascicle elongation, to the total lengthening of the MTU during stretching (Figure 5).

In pre-training, at 25° of ankle dorsiflexion, the average Lf.cos µ was 11.6 ± 2.6 mm, which corresponded to 66.4 ± 15.3% of the total lengthening of the MTU. This means that the average Lt was 33.6 ± 15.3%, or 5.9 ± 2.8 mm, of the increase in the L_MTU_.

In post-training, at the same angulation of 25°, the average Lf.cos µ was reduced to 9.5 ± 2.7 mm, or 54.2% (45.8 ± 14.3%) of the total lengthening of the MTU. Therefore, the average Lt increased to 45.8% (54.2 ± 14.3%), or 8.0 ± 2.5 mm of the total increase in the L_MTU_.

## 4. Discussion

As expected, the results of the current study show that an intermittent passive stretch training protocol during 5 weeks of the plantar flexor muscles increased the maximum ankle dorsiflexion and reduced the relative passive torque produced by the MTU (15° to 25° dorsiflexion) in healthy subjects. These changes showed a decrease in ankle passive stiffness and dissipated elastic energy. Architectural parameters (pennation angle and fascicle length) showed a significant difference between the slopes of their evolution suggesting a modification in the mechanical behavior after stretch training.

### 4.1. Changes in Passive Torque

Many studies have demonstrated that the maximum ROM can be increased after a stretch training protocol [1,2,4,9,35]. However, Toft et al. [7] suggested that passive torque measurements would be more objective than the range of motion measurements, due to the fact that psychological factors do not interfere with the results. Therefore, in the present study, passive torque measurements and maximum ROM were utilized to assess flexibility. The stretch training increased the maximum ankle dorsiflexion and the associated maximum passive torque by ±13%. The greater maximum passive torque has been attributed to a greater tolerance to stretch [12,36]. The increase in stretch tolerance could be linked to a change in the afferent input from nociceptive nerve endings and mechanoreceptors [12,37].

Consistent with previous in vivo studies, the passive torque produced by the plantar flexor muscles increased exponentially with progressive ankle dorsiflexion [6,16,18,20,36]. After the stretch training, we observed a significant reduction in the relative passive torque for ankle angles of 15–25° reaching 27.9 ± 6.2 Nm at 25°. Kubo et al. [24] and Toft et al. [7] also reported a decrease in passive torque after a three-week stretch training protocol.

The passive stiffness values, calculated between 15° and 25°, reached an average of 1.34 ± 0.5 Nm/° before stretch training, which is comparable to the value of 1.43 ± 0.3 Nm/° reported by Kubo et al. [24]. After the stretch training, the passive stiffness values decreased significantly by 21.7%, to reach the value of 1.04 ± 0.4 Nm/°. In contrast, Kubo et al. [24] demonstrated a decrease of 13.4% after twenty consecutive days of stretching and reported a value of 1.24 ± 0.3 Nm/°. The present results concur with previous studies, in which decreased muscle stiffness [27,35,38,39,40] has been observed after static stretching. In contrast, other studies did not find changes in muscle stiffness [41]. The discrepancy can be due to different training modalities, such as intensity, frequency, number of exercises, and overall duration [3,4,40]. The myotendinous stiffness could be related to the intrinsic stiffness of muscles, tendons, and connective tissues surrounding the whole MTU but also to neural mechanisms [35,40,42,43].

The hysteresis loop, produced by the loading and unloading curves of passive torque as a function of the ankle angle, enabled us to determine the dissipated elastic energy. In our study, the dissipated elastic energy decreased by −61.2% (38.8 ± 18.6%; *p* < 0.001) after the stretch training. This result is higher than the decrease of approximately 37% observed by Kubo et al. [24]. These results will explain the higher degree of flexibility in the MTU obtained after the same intermittent passive stretch training [44]. This finding is of interest since it shows better storage of elastic energy, which was then converted to kinetic energy during the push-off phase of the drop jump [44]. However, the mechanisms that resulted in the decrease in hysteresis remain ambiguous. Nevertheless, the cyclic strain with a repetitive passive motion is considered to cause a rapid redistribution of polysaccharides and water within the collagen framework leading to changes in muscle thixotropy and viscosity [9,45], which can explain our results.

### 4.2. Changes in Muscle Architecture

In agreement with previous studies [17,20,21,32], muscle architecture changed during passive stretching: Fascicle length increased and pennation angle decreased in the MG muscle when the ankle angle was rotated from 0° to 25°. These changes in MG architecture contributed to the increase in the whole MTU length during passive muscle stretching.

Statistical analysis of the pre- and post-test results of the linear regression of pennation angle and fascicle length showed a significant difference between the slopes (ANCOVA *p* < 0.001) in a neutral position. The pennation angle decreased linearly by −7.5% (92.5 ± 1.4%), and the L_f_ increased linearly by +16.0% (84.0 ± 3.9%) during ankle dorsiflexion. More recent studies did not report changes in the muscle architecture of MG after 4 to 12 weeks of static stretch training [1,2,9]. Nevertheless, Freitas and Mil-Homens [23] reported a significant increase in the fascicle length of the biceps femoris following an 8-week high-intensity stretch training program. These studies demonstrate that, in the case of the hamstring, a static stretching program may change the muscle architecture, which may result in increased strength or performance. These results suggest that the effects of static stretch training on muscle strength and architecture may differ on the basis of the target muscle and total exercise. Our contrasting results may be understood because very seldom studies followed the architectural behavior of the MTU measured during progressive stretching, allowing researchers to unravel some otherwise unseen changes.

In addition to the intrinsic stiffness of the muscle, the MTU resting passive torque can also be partly induced by neural mechanisms [6,11,25,46]. By reducing passive resistance due to tonic reflex activity, the passive stretch training protocol may have contributed to a greater elongation of the muscle fibers through greater muscle relaxation in a neutral position.

### 4.3. Estimation of the Relative Contribution of Fascicles and Tendon

To calculate the change in L_MTU_ during each stretching angle, the regression equation provided by Grieve et al. [34], as introduced previously, was used. Our results, which matched those of previous studies [32], indicated that L_MTU_ increased by 4.4% (17.5 ± 1.2 mm) during ankle dorsiflexion to reach an average length of 414.0 ± 27.8 mm at 25°.

Moreover, to determine the relative contributions of the fascicle and free-tendon elongation to the total lengthening of the MTU during stretching, it was necessary, first, to determine the change in Lf along the longitudinal axis of the MTU by multiplying Lf with the cosine of the pennation angle; and secondly, to calculate the change in Lt by subtracting the change in the Lf.cos µ from the change in L_MTU_. In our study, before the stretch training, Lf.cos µ and Lt increased during passive ankle dorsiflexion by 11.6 ± 2.6 mm and 5.9 ± 2.8 mm, respectively. Our results confirm the reported changes in the original paper of Mizuno [47]. Therefore, before the stretch training, the relative fascicle elongation contributed to 66.4 ± 15.3% of the total lengthening of the MTU, and the relative free tendon contributed to 33.6 ± 15.3% of the increase in the L_MTU_.

The relative contributions of the fascicle and free-tendon elongation remain disputed in the literature. Indeed, although Abellaneda et al. [32] reported a fascicle elongation contribution slightly greater than our results (71.8%), the general behavior remained the same, with fascicle elongation contributing more than the elongation of the free tendon. This is contrary to the results of Herbert et al. [17], who showed a free-tendon contribution of ≈75%. Other studies, such as Morse et al. [20], reported a similar contribution of fascicule and free-tendon elongation changes to the whole MTU lengthening. These variations could be explained by differences in the experimental methodology. Herbert et al. [17] conducted their study with the knee flexed, contrary to the study of Abellaneda et al. [32] and our study, both of which kept the leg straight. Moreover, Morse et al. [20] estimated the relative contributions with a different method. In their study, the authors measured the changes in the free tendon directly and inferred the changes in muscle (fascicles and aponeurosis) length.

After the training, at 25° of dorsiflexion, a change in the free-tendon and fascicle elongation was observed. The contributions of fascicle elongation and the free-tendon elongation balanced each other out, as indicated by the contribution values of 54.2 ± 14.3% and 45.8 ± 14.3%, respectively, of the total lengthening of the MTU.

### 4.4. Limitations

There were some limitations in this study. First, we did not have a control group. In addition, supervision of stretching sessions was not carried out on weekends. Furthermore, it is possible that the six men in the study biased the results given their significant stiffness. Thus comparing the mechanical and architectural parameters behavior between males and females should be considered. It is also possible that their feelings and stretch tolerance may be different. Another limitation is that other neuromuscular characteristics (average electromyography and maximum voluntary contraction) were not measured. Finally, it is possible to have underestimated the stretching time since the students also stretched after the sport class although not in a specific way.

## 5. Conclusions

In conclusion, the five-week intermittent passive stretch training protocol produced viscoelastic and architectural changes in the human MTU. Our protocol induced changes in the relative contributions of the free tendon and fascicles. Furthermore, the stretch training modified the passive torque–ankle angle relationship and decreased the dissipated elastic energy. This modification could, over time, influence the athletes’ performance during stretch-shortening cycle exercises. Further investigations are required to elucidate this point.

## Data Availability

Data are available at request from the authors.
